# Characteristics of goal-setting tools in adult rehabilitation: A scoping review

**DOI:** 10.1177/02692155231197383

**Published:** 2023-08-30

**Authors:** Yuho Okita, Yuko Kawaguchi, Yuki Inoue, Kanta Ohno, Tatsunori Sawada, William Levack, Kounosuke Tomori

**Affiliations:** 1 School of Health Science, Swinburne University of Technology, Melbourne, Australia; 2 Department of Rehabilitation, 469534 Kaikoukai Rehabilitation Hospital, Aichi, Japan; 3 Central Rehabilitation Department, 84178 Yokohama Rousai Hospital, Kanagawa, Japan; 4 Major of Occupational Therapy, Department of Rehabilitation, School of Health Sciences, 201117 Tokyo University of Technology, Tokyo, Japan; 5 Department of Medicine, University of Otago, Wellington, New Zealand

**Keywords:** Goal setting, rehabilitation, scoping review, goal-setting tool, shared decision-making

## Abstract

**Objectives:**

This scoping review aims to map the literature on goal-setting tools in adult rehabilitation, exploring their characteristics, target users and supporting evidence to inform practice and future research in this area.

**Methods:**

We completed a comprehensive search of four databases to identify relevant articles on tools for goal setting in rehabilitation. We followed Arkey and O’Malley's scoping review process to guide article selection, data extraction and data analysis.

**Results:**

We identified a total of 165 studies that reported on 55 different goal-setting tools, including tools for goal selection and goal documentation (*n* = 31), goal setting and intervention planning (*n* = 15), and for measuring the quality of the goal-setting process (*n* = 9). Over half of the tools were primarily designed for use in rehabilitation of physical disabilities (*n* = 32). Some tools fell under multiple sub-categories based on their characteristics as follows: 22 framework tools, 12 interview tools, 9 outcome measurement tools for goal achievement, 6 outcome measurement tools for goal quality and 25 documentation tools. The majority of goal-setting instruments targeted goals at the level of activity and participation (*n* = 51) and aimed to facilitate a client-centred or shared decision-making approach to rehabilitation planning (*n* = 46).

**Conclusions:**

This study provides a comprehensive overview of existing goal-setting tools, highlighting their characteristics, target users and identified needs. These findings can enhance practitioners’ awareness of the range of goal-setting tools available and can enable more effective utilization of these tools in clinical practice. Further research should investigate how clinicians can combine multiple tools to deliver goal setting.

## Introduction

Clinical practice guidelines usually advocate for the integration of goal setting in the rehabilitation process.^1–3^ Goal setting is thought to enhance clients’ motivation and engagement in rehabilitation and to improve communication and collaboration between healthcare practitioners and clients, ultimately leading to accelerated recovery and better health outcomes.^4,5^ Various approaches have been proposed to increase the involvement of rehabilitation clients in the goal-setting process in order to facilitate shared decision-making.^6,7^ However, certain barriers still impede application of collaborative goal setting in some practice areas. These barriers include challenges around understanding and meeting clients’ information needs,^7^ limitations in clients’ communication or cognitive abilities, and limitations in therapists’ skills or knowledge to apply goal setting effectively.^4^ Notably, following an audit of goal-setting practice across seven rehabilitation wards, Saito et al.^8^ found that 79% of goals set by occupational therapists did not align with clients’ perceived goals. This study, and others like it,^10,184^ suggests that there remains a need for further investigation into the best tools to facilitate therapist communication and client engagement in the goal-setting process.^8^

In this study, the term ‘goal-setting tool’ is used to refer to a specific test, guide, interview, questionnaire, or other structured method of clinical interaction, which is designed to support the goal-setting process or to evaluate outcomes arising from it. Goal-setting tools may aid in problem identification, shared goal prioritization, treatment progress monitoring, measurement of goal achievement, or active client involvement. Previous systematic reviews have suggested that tools designed to aid client involvement in clinical processes can enhance the meaningful involvement of older adults in clinical decision-making and rehabilitation clients in individualized, person-centred goal setting – although the choice of which tool is best to use in which setting remain somewhat unclear.^9^ Indeed, although various goal-setting tools have been developed,^11,12^ there is a lack of comprehensive reviews detailing their characteristics and supporting their application in clinical practice. The objective of this scoping review therefore was to identify all existing goal-setting tools and to categorize their key features in order to guide future research on the development of effective strategies and tools for goal setting in clinical practice.

## Methods

This scoping review followed Arksey and O’Malley’s framework,^13^ which comprises a five-step process as follows: (a) identification of review questions; (b) identification of relevant studies; (c) selection of studies; (d) data extraction; and (e) summarization and reporting of results. The reporting of this review adheres to the Preferred Reporting Items for Systematic Reviews and Meta-Analyses – Extension for Scoping Reviews checklist. The completed forms are attached in Supplemental material 1.^14^

The aim of this scoping review was to address the following four research questions related to the use of goal-setting tools in adult rehabilitation as follows:

1. What tools have been developed to support or enhance goal setting in adult rehabilitation?
2. How are these goal-setting tools used and in which contexts?
3. What are the characteristics of these goal-setting tools?
4. What future research needs to be conducted to better understand the impact of these goal-setting tools on the quality of goal setting and on improved health outcomes?

We included all studies published in English that reported on any tools used to support goal setting in adult (>18 years) rehabilitation. Rehabilitation, in this context, refers to strategies or interventions aimed at mitigating the effects of impairments, activity limitations or participation restrictions in individuals with various health conditions.^28^ To be included in the review, articles needed to focus on tools specifically designed to assist in different aspects of goal setting, including goal selection, goal documentation, planning or pursuit of goal achievement, measurement of goal attainment, or assessment of the quality of the goal-setting process. We excluded studies that only used activity monitoring or step counts as a goal of therapy and studies that investigated intervention programs, technologies, websites or other applications primarily designed for purposes other than goal setting, but with minor goal-setting features. Furthermore, we excluded papers that primarily focused on describing theories underpinning goal setting. All articles, except for study protocols, conference abstracts, book chapters and theses, were eligible for inclusion. There were no exclusions based on methodological quality assessment, and no restrictions were placed on the publication year.

We conducted a comprehensive literature search of four databases, Scopus, PubMed, MEDLINE and ProQuest, from inception to 28 February 2023. The search involved identifying articles which used three main terms (‘tool’, ‘goal’ and ‘rehabilitation’), synonyms of these terms, or other related terminology (see Supplemental material 2 for details). To ensure the inclusion of appropriate studies, two or more authors independently reviewed each title and abstract to exclude duplicate or irrelevant studies. We subsequently retrieved all full-text publications, and two authors independently screened the full text for inclusion, identifying and recording reasons for exclusion of ineligible studies. We resolved any disagreements through discussion or, when required, consultation with a third review author.

Two authors (YO and KT) collaboratively developed a data extraction template, which was further refined through discussions with the other authors. Using this template, all authors contributed to extracting data from assigned articles. Both YO and KT reviewed the accuracy of the extracted information from all assigned articles. Any discrepancies or differences in extracted data were resolved through discussions involving at least two authors. We extracted data on both the characteristics of the included study and on the characteristics of the goal-setting tools. For the study characteristics, we collected information on the country of the first author’s affiliation, study design, study setting, sample size and patient population. Regarding the goal-setting tools, we collected data on the name of the identified tool, intended user, intended patient population, intended purpose, type of tool, intended domain of goals (i.e. body function or structure, activity or participation or environmental factors), type of decision-making involved, description of the tool and study findings.

We grouped each goal-setting tool into one of three categories as follows:

- Tools for goal selection and documentation: These were tools specifically designed to facilitate the goal-setting process, which had a primary focus on setting goals but which sometimes included elements that could be used as outcome measures. The main aim of these tools was to support the identification and documentation of goals.
- Tools for goal setting and intervention delivery: These were tools that had been designed to facilitate the planning and development of goal-directed interventions. While these tools also could sometimes support the goal-setting process, their main focus was on enabling and guiding interventions that were aligned with the identified goals.
- Tools for measuring the quality of goal setting: These were tools that were designed to primarily focus on evaluating the quality of goal setting. They are intended to help assess the effectiveness and appropriateness of the goal-setting process from the perspective of therapists, clients, or other stakeholders.

We then grouped each goal-setting tool across a range of sub-categories. These sub-categories were developed iteratively by the two lead authors (YO and KT) following evaluation and discussion of the preliminary review findings, and were not mutually exclusive – in other words, a single approach to goal setting could be positively identified as belonging to any number of these sub-categories. These additional sub-categories of goal-setting tools were as follows:

- Goal-setting frameworks: This sub-category referred to a structured approach or model that provided guidance for users to follow a specific way of goal setting. This included step-by-step guides to goal setting and goal-setting approaches based on principles of engagement with clients.
- Interview tools: Interview tools were resources or instruments that facilitated or guided semi-structured interviews in order to assist with gathering information relevant to the goal-setting process.
- Outcome measures (goal achievement): Tools under this sub-category allowed users to measure goal attainment or achievement. These tools assessed the extent to which the goals had been accomplished or the desired outcomes had been realized.
- Outcome measures (goal-setting quality): Tools under this sub-category enabled users to measure the quality of goal setting or other aspects related to the goal-setting process. These tools might be used to evaluate the effectiveness, appropriateness, or value of the goal-setting process or of the goals themselves. They were designed to provide insights into the quality or characteristics of the goals set.
- Documentation tools: This sub-category of tools included any physical or digital resource, excluding information and communications technology, that enabled the user to take notes for goal setting or to record goals that were set. Examples included paper-based forms, templates or digital documents specifically designed for documenting goals.
- Information and Communications Technology tools: This sub-category referred to any tools that used digital technology to enable the storage, retrieval, manipulation, transmission, or reception of information. In the context of goal setting, Information and Communications Technology tools include digital platforms, software, applications or devices that had functionalities for goal setting.

Regarding the target patient population, we grouped each tool into the one of four broad categories of medical conditions as follows:

- Physical disability: Tools designed for individuals with physical disabilities that affect their physical condition, such as mobility, physical capacity, range of motion and strength (e.g. brain injury, spinal cord injuries, cerebral palsy, cardiovascular disabilities and visual impairments).
- Psychiatric disability: Tools aimed at individuals with psychological problems that impacted their mental well-being, including their thoughts, emotions and behaviours (e.g. depression, eating disorders, schizophrenia and bipolar disorder).
- Age-related disability: Tools intended for use with individuals who are described as ‘older’ or ‘elderly’ or are aged over 65 years old. These tools were specifically tailored to address the unique needs and challenges associated with aging.
- Others/specified condition: Tools that were specifically designed for addressing the needs of individuals with a single particular medical condition (e.g. aphasia, pain, or cancer).

We also extracted data on the target domain of the goal-setting process (body structure or body function, activity or participation, environmental factors, or non-specific – according to the International Classification of Functioning, Disability and Health categories),^15^ the type of decision-making involved (e.g. client-centred or shared decision-making; therapist-led, caregiver-centred, or unclear), and the intended users of the tool (e.g. physiotherapy, occupational therapy, physician or psychologist, other healthcare professional, or clients or caregiver). While a scoping review does not typically require an in-depth analysis of specific evidence types, we wanted to map out the spread of evidence supporting the effectiveness of different goal-setting tools to achieve improvements in either the rehabilitation process (e.g. degree of client engagement) or health outcomes. In order to report on the highest quality evidence of effectiveness, we focused on studies that used randomized control trial designs. The aim of this aspect of the analysis was to evaluate the spread of existing evidence on the effectiveness of these tools and to identify gaps in the evidence base to guide future research.

## Results

**Figure 1.**
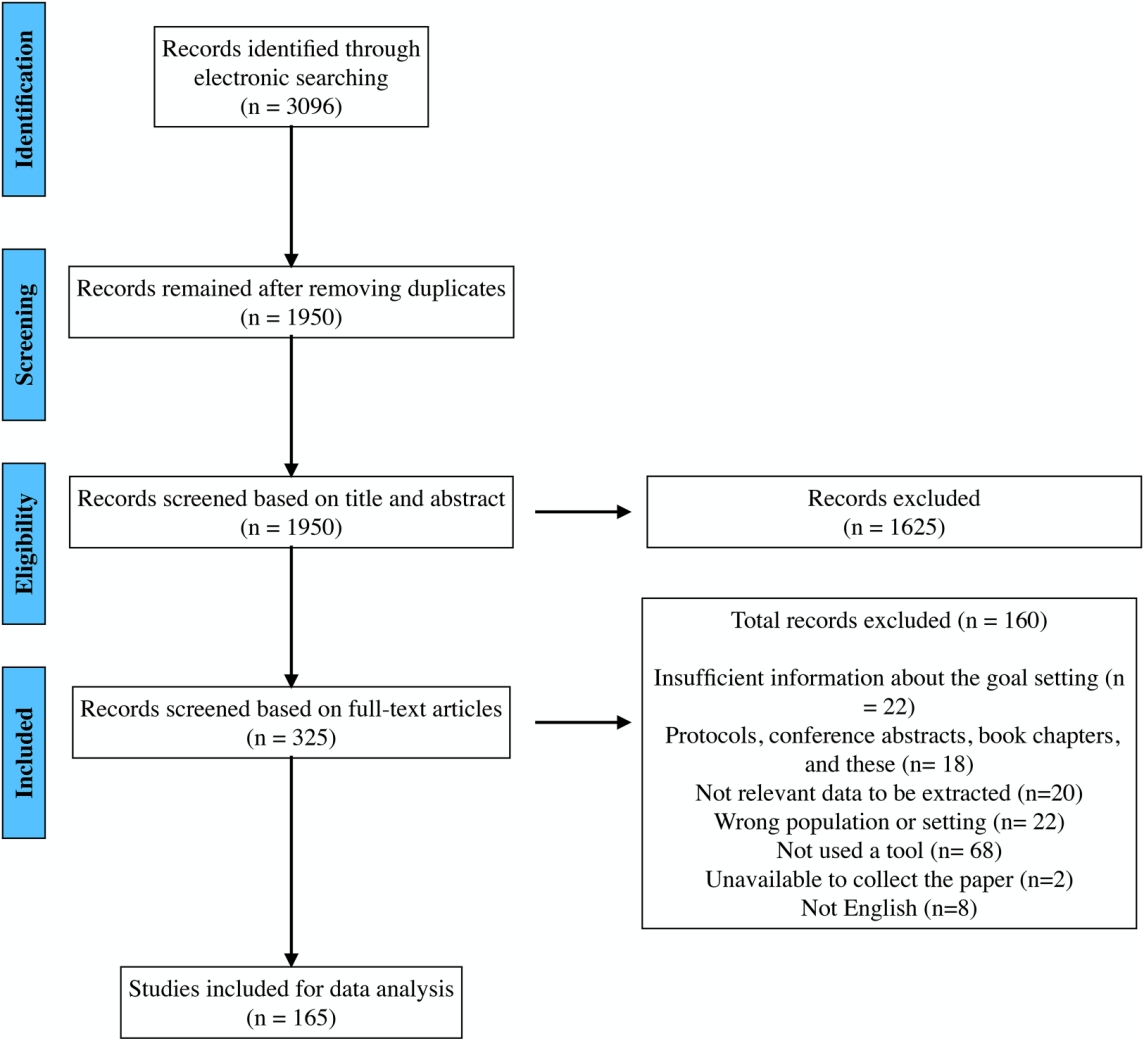
Preferred Reporting Items for Systematic Reviews and Meta-Analyses (PRISMA) flow diagram displays article selection process.

The initial search yielded a total of 1950 records, which were narrowed down to 165 full text articles for final analysis. The screening process and reasons for exclusions are reported in Figure 1. The majority of the articles (119/165) were published in the last decade. The identified study designs included 47 observational studies, 26 qualitative studies, 21 review papers (including narrative reviews, literature reviews and scoping reviews), 16 randomized controlled trials, 12 case studies, 11 systematic reviews, 10 mixed methods studies, 2 surveys, 14 articles with unclear designs and 6 other research designs (such as simulated studies, prospective descriptive studies, comparative designs, clinical audits, non-controlled interventions and practical guides).

**Figure 2.**
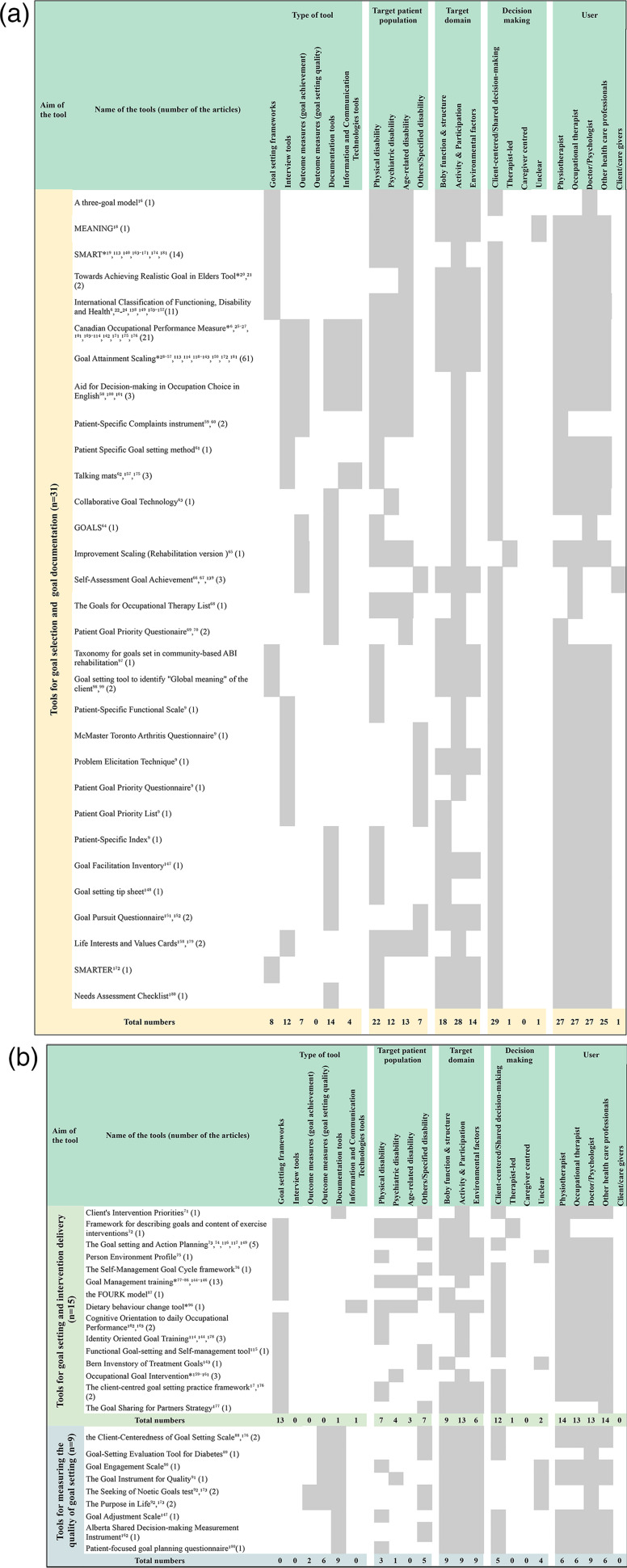
(a and b) Identified goal-setting tools with their respective characteristics.

Identified goal-setting tools along with their respective characteristics are shown in Figure 2 (a detailed description of each tool can be found in Supplemental material 3). A total of 55 distinct goal-setting tools were identified among the 165 articles.

### Characteristics of the identified goal-setting tools

Of the 55 identified goal-setting tools, 56% (31/55) were tools for goal selection and goal documentation, and 27% (15/55) were for goal setting and intervention delivery, while the remaining 16% (9/55) were for measuring the quality of goal setting. These tools were tested across various patient populations, including those with as follows: physical disabilities (32/55), psychiatric disabilities (17/55), age-related disabilities (16/55) and other/specific medical conditions (19/55). The majority of the identified tools appeared to be not specifically intended for use by a particular health profession.

Six of the identified tools were described and studied in five or more articles. These included Goal Attainment Scaling including Goal Attainment Scaling for Major Depressive Disorder and Goal Attainment Scaling for Upper Limb Spasticity^28–57,113,114,118–143,150,172,181^ (*n* = 61), Canadian Occupational Performance Measure^6,25–27,101,103–114,142,171,175,176^ (*n* = 21), Goal Management Training^77–86,144–146^ (*n* = 13), International Classification of Functioning, Disability and Health^6,22–24,138,149,153–157^ (*n* = 11) and the Goal setting and Action Planning^73,74,116,117,149^ (*n* = 5). Interestingly, none of the identified tools intended for measuring the quality of goal setting were supported by more than two research studies, potentially indicating limited evidence for such tools.

Many of the identified tools met the criteria for multiple sub-category types, but included as follows: 22 goal-setting frameworks, 12 interview tools, nine outcome measures for goal achievement, six outcome measures for goal-setting quality and 25 documentation tools. The majority of the tools categorized as being for goal setting and intervention delivery appeared to be framework tools, whereas no framework tools were identified in the tools categorized as outcome measures of goal-setting quality.

Only five of the identified tools made use of information and communications technology for goal setting, four of which were categorized as tools for goal selection and goal documentation. These included Canadian Occupational Performance Measure,^6,25–27,101,103–114,142,171,175,176^ Goal Attainment Scaling,^28–57,113,114,118–143,150,172,181^ Aid for Decision-making in Occupational Choice in English^58,100,101^ and Talking mats.^62,157,175^ Of note: Goal-setting tools that included an Information and Communications Technology format appeared to be primarily designed for use in an in-person context and not specifically for remote use (i.e. for rehabilitation services provided by telerehabilitation).

The majority of tools targeted goals set at the level of activity and participation (51/55) and involved a client-centred or shared decision-making approach to goal selection (46/55). Some tools supported goals being set at the level of body function or body structure (36/55) or which targeted environmental factors (29/55). A minority of tools (2/55) enabled a therapist-led approach to goal setting. There were no tools specifically designed for caregiver-centred decision-making.

### Research evidence on the effectiveness of goal-setting tools

Of the identified 55 goal-setting tools, only seven had been studied via randomized control trial methods, indicated by an asterisk (*) in Figure 2. The tools supported by randomized control trials included as follows: Goal Management Training (*n* = 6)^77,79,81,83,85,86^; Goal Attainment Scaling (*n* = 4)^36,57,126,130^; SMART (*n* = 2)^163,170^; Towards Achieving Realistic Goal in Elders Tool (*n* = 1)^21^; Dietary Behaviour Change Tool (*n* = 1)^96^; Canadian Occupational Performance Measure (*n* = 1)^108^; and Occupational Goal Intervention (*n* = 1).^160^ The majority of research providing evidence to support use of the identified tools involved non-randomized control trial design or observational methods, indicating lower levels of evidence regarding the effectiveness of the approach. One of the tools, Goal Attainment Scaling, was only used to measure outcomes in randomized control trials rather than using randomized control trial methods to test the effectiveness of the tool to improve the rehabilitation process or patient outcomes.^36,57,126,130^ Two other tools, Canadian Occupational Performance Measure^108^ and SMART,^163,170^ were used as part of a goal-direct approach to rehabilitation (i.e. for goal-directed cognitive rehabilitation,^108^ goal-directed interval walking training,^163^ and goal-directed low back pain rehabilitation^170^) so did not provide evidence of the effectiveness of goal setting in isolation of the rest of a comprehensive rehabilitation programme. The remaining identified tools (Goal Management Training^77,79,81,83,85,86^; Towards Achieving Realistic Goal in Elders Tool^21^; Dietary Behaviour Change Tool^96^; and Occupational Goal Intervention^160^) all investigated the effectiveness of an approach to goal setting on changes in one or more health outcomes compared to a control group that did not receive goal setting.

Among the randomized control trials that investigated the goal-setting tools as primary interventions compared to control groups, Parsons et al.^21^ evaluated the impact of Towards Achieving Realistic Goal in Elders Tool, a goal facilitation tool, on health-related quality of life, social support and physical function in community-dwelling older people receiving home care. The study concluded that the use of Towards Achieving Realistic Goal in Elders Tool led to significant improvements in health-related quality of life in the intervention group and helped individualize activities tailored to the participants’ needs and goals.

Several randomized control trials have investigated the effectiveness of Goal Management Training to address problems with executive functioning among different populations.^77–86^ For example, randomized control trials supported the effectiveness of Goal Management Training alone in improving executive functioning and daily activity performance in patients with spina bifida^83^ and chronic acquired brain injury.^81^ Other studies supported the effectiveness of combining Goal Management Training with other interventions to improve executive functioning.^79^ These included a randomized control trial by Casaletto et al.^77^ showing that a combination of Goal Management Training and metacognitive training had significant effects on everyday multitasking and metacognitive performance. Similarly, Levine et al.^79^ compared an expanded version of Goal Management Training to another intervention, Brain Health Workshop, among patients recovering from brain disease, and found that Goal Management Training was efficacious in the rehabilitation of executive function. Additionally, a randomized control trial by Bertens et al.^85^ supported the effectiveness of Goal Management Training combined with errorless learning for training complex daily tasks in brain-injured patients with executive dysfunction.

## Discussion

This scoping review aimed to increase awareness of available goal-setting tools in rehabilitation and to provide insights into their characteristics in order to assist practitioners to utilize them more confidently and effectively in clinical practice. This discussion will focus on the three categories of goal-setting tools as follows: (a) tools for goal selection and goal documentation; (b) tools for goal setting and rehabilitation planning; and (c) tools for measuring the quality of goal setting.

In the category of tools for goal selection and goal documentation (see Figure 2(a)), several frameworks were identified that can enhance understanding of goals and goal setting. These goal-setting tools help improve the clarity and focus of goal setting by providing theoretical guidance and by highlighting important factors to consider. They can save time and simplify the goal-setting process. Furthermore, seven of these tools (see Figure 2(a)) not only support the goal-setting process but also can be used as outcome measures to evaluate goal achievement. These tools can support evidence-based intervention delivery and enhance the therapy planning process.^95^ The majority of identified goal-setting tools are applicable across various medical conditions and target activity and participation domains. These tools facilitate client-centred or shared decision-making approaches and can be easily integrated into clinical practice without extensive training. Additionally, four of these tools (see Figure 2(a)) are available in a digital format, which may make them easier for health professionals to integrate into practice. The use of Information and Communications Technology in goal setting may facilitate client engagement, providing a platform for expressing ideas and opinions about goals, establishing collaborative structures, and managing the complexity of goal setting.^12^

In the category of tools for goal setting and intervention delivery (see Figure 2(b) upper), the majority of tools (13/15) were identified as frameworks designed to guide the integration of goal setting into rehabilitation planning. These frameworks offer theories and evidence-based methods that support the participants to work on goal-directed therapy by facilitating goal-directed behaviours. For example, Goal Management Training^77–86,144–146^ is a framework that involves a five-stage process to address impairments in a person's capacity to engage in goal-directed behaviour. Similarly, Goal Setting and Action Planning^73,74,116,117,149^ is designed to support informed decision-making about goal adjustment or goal disengagement by undertaking four stages as follows: (a) goal negotiation and setting; (b) action planning and coping planning; (c) action; and (d) appraisal, feedback and decision-making. It has been reported that many health professionals find it challenging to negotiate with clients and family members around the selection of realistic, achievable and personally meaningful goals.^90^ By incorporating these goal-setting frameworks into clinical practice, therapists can promote goal-directed behaviours and strengthen their therapeutic alliance with clients, with the ultimate aim of improving rehabilitation outcomes.

In the category of tools for measuring the quality of goal setting (see Figure 2(b) lower), nine different tools were identified. These tools provide valuable insights into the goal-setting process and can contribute to improving the quality of goal setting in rehabilitation practice. One example is the Client-Centredness of Goal Setting scale,^88,176^ which was specifically designed to help therapists assess the extent to which goal setting aligns with client-centred principles, facilitating a collaborative and client-driven approach to goal setting. By incorporating the client’s input and incorporating their responses into the goal-setting process, therapists can ensure that goals are meaningful and tailored to the individual’s needs and preferences. Other tools (see Figure 2(b) lower) offer additional dimensions for evaluating the quality of goal setting. These tools assess various aspects of goal setting, including goal engagement, the pursuit of meaningful goals, the alignment of goals with personal values and the effectiveness of shared decision-making. This evaluation process can inform the therapeutic process, improve the client’s experience and optimize goal attainment and overall rehabilitation outcomes.

While this scoping review provides valuable insights into goal-setting tools in rehabilitation, there are several limitations that need to be acknowledged. One limitation is that this review was not designed to investigate the psychometric properties of the identified tools. Validity and reliability assessments are crucial for ensuring the effectiveness and applicability of these tools in clinical practice. Future research should focus on evaluating the psychometric properties of tools that have been developed to measure rehabilitation outcomes or the quality of goal-setting processes. Another limitation is the exclusion of grey literature (i.e. conference papers or research theses) from this review, which may have resulted in the omission of relevant information on the most recent goal-setting tools. Additionally, this review focused only on English-language tools, so potentially missed information about goal-setting tools that have been developed in other languages. Despite these limitations, this scoping review lays the groundwork for future research in the field of goal-setting tools in rehabilitation. The identified tools offer clinicians a starting point for selecting appropriate tools based on their specific needs and objectives. Further research should explore the combination of different goal-setting tools to address the diverse needs of patient populations in various settings. By understanding how different goal-setting tools can be used together for greatest effect, clinicians can develop comprehensive and tailored approaches to goal setting in rehabilitation, with the ultimate aim of improving person-centred rehabilitation outcomes.

**Clinical messages**

With over 50 goal-setting tools available, clinicians can select from a wide range of approaches to meet the specific needs of their area of clinical practice.

- Goal-setting tools facilitate collaborative goal setting, measure therapy outcomes and track progress.
- The right goal-setting tools can support planning, delivery and evaluation of rehabilitation interventions.
- Understanding the characteristics and quality of goal-setting tools can help selection of the right tool for the right client in the right rehabilitation context.

## Supplemental Material

sj-docx-1-cre-10.1177_02692155231197383 - Supplemental material for Characteristics of goal-setting tools in adult rehabilitation: A scoping review Click here for additional data file. Supplemental material, sj-docx-1-cre-10.1177_02692155231197383 for Characteristics of goal-setting tools in adult rehabilitation: A scoping review by Yuho Okita, Yuko Kawaguchi, Yuki Inoue, Kanta Ohno, Tatsunori Sawada, William Levack and Kounosuke Tomori in Clinical Rehabilitation

sj-docx-2-cre-10.1177_02692155231197383 - Supplemental material for Characteristics of goal-setting tools in adult rehabilitation: A scoping review Click here for additional data file. Supplemental material, sj-docx-2-cre-10.1177_02692155231197383 for Characteristics of goal-setting tools in adult rehabilitation: A scoping review by Yuho Okita, Yuko Kawaguchi, Yuki Inoue, Kanta Ohno, Tatsunori Sawada, William Levack and Kounosuke Tomori in Clinical Rehabilitation

sj-pdf-3-cre-10.1177_02692155231197383 - Supplemental material for Characteristics of goal-setting tools in adult rehabilitation: A scoping review Click here for additional data file. Supplemental material, sj-pdf-3-cre-10.1177_02692155231197383 for Characteristics of goal-setting tools in adult rehabilitation: A scoping review by Yuho Okita, Yuko Kawaguchi, Yuki Inoue, Kanta Ohno, Tatsunori Sawada, William Levack and Kounosuke Tomori in Clinical Rehabilitation
