# Supplementary material for: Characteristics of goal-setting tools in adult rehabilitation: A scoping review
Source: Clin Rehabil. 2023 Aug 30;38(2):234–50. doi: 10.1177/02692155231197383 (PMC10725121; doi:10.1177/02692155231197383)
Supplement: sj-docx-2-cre-10.1177_02692155231197383 - Supplemental material for Characteristics of goal-setting tools in adult rehabilitation: A scoping review [file sj-docx-2-cre-10.1177_02692155231197383.docx]

Supplementary material 2. The search strategy for all databases

PubMed

(rehabilitation[tw] or "occupational therap*"[tw] or "physical therap*"[tw] or physio*[tw] or

"speech therap*"[tw] or "speech patholog*"[tw]) AND (goal*[ti] or "goal setting"[ti] or "goalsetting"[

ti] or "goal-oriented") AND (web[tw] or "Mobile Applications"[Mesh] or

Technology[tw] or device[tw] or "approach"[tw] or tool[tw] or instrument[tw])

MEDLINE/CINAHL

Full text only

(rehabilitation or "occupational therap*" or "physical therap*" or physio* or "speech therap*" or

"speech patholog*") AND (goal* or "goal setting" or "goal-setting" or "goal-oriented") AND

(web or "Mobile Applications" or Technology or device or approach or tool or instrument)

Scopus

( TITLE-ABS-KEY ( web OR "Mobile Applications" OR technology OR device OR approach

OR tool OR instrument ) AND TITLE ( goal* OR "goal setting" OR "goal-setting" OR "goaloriented"

) AND TITLE-ABS-KEY ( rehabilitation OR "occupational therap*" OR "physical

therap*" OR physio* OR "speech therap*" OR "speech patholog*" ) )

Proquest

FT(web or "Mobile Applications" or Technology or device or approach or tool or instrument)

and TI(goal* or "goal setting" or "goal-setting" or "goal-oriented") and FT(rehabilitation or

("occupational therapies" OR "occupational therapist" OR "occupational therapists" OR"occupational therapy") or ("physical therapies" OR "physical therapist" OR "physical

therapists" OR "physical therapy") or physio* or ("speech therapist" OR "speech therapists" OR

"speech therapy") or ("speech pathologist" OR "speech pathologists" OR "speech pathology"))
