# Supplementary material for: Characteristics of goal-setting tools in adult rehabilitation: A scoping review
Source: Clin Rehabil. 2023 Aug 30;38(2):234–50. doi: 10.1177/02692155231197383 (PMC10725121; doi:10.1177/02692155231197383)
Supplement: sj-pdf-3-cre-10.1177_02692155231197383 - Supplemental material for Characteristics of goal-setting tools in adult rehabilitation: A scoping review [file sj-pdf-3-cre-10.1177_02692155231197383.pdf]

Supplementary material 3. Description of all the identified goal-setting tools

Aim of the tool	Name of the tools	Description
**Tools for goal selection and goal documentation (n=31)**	A three-goal model[16] (1)	The three-goal model helps understand three types of goals: (1) Disease specific or symptom specific goals; (2) Functional goals; (3) Fundamental goals be identified as a guide to clinical care of patients with multiple-long term conditions. This could help countribute in making daily care more patient-goal oriented.
	MEANING[18] (1)	MEANING (M: Meaning, E: Engage, A: Anchor, N: Negotiate, I: Intention-implementation gap, N: New goals, G: Goal as behaviour change) is a key term and as an acronym to underpin, remind and support rethinking actions and activities in goal setting. This is to be a practical tool to support a theoretically informed focus in goal setting.
	SMART*[19,113,140,163–171,174,181] (14)	SMART (A novel approach to writing specific, measurable, achievable, realistic/ relevant and timed (SMART) goals is a key term and as an acronym to support goal setting more easily and quickly.
	Towards Achieving Realistic Goal in Elders Tool*[20,21] (2)	The use of Towards Achieving Realistic Goal in Elders Tool follows recognised fundamental principles of the goal-setting process (i) goal identification, (ii) goal negotiation, (iii) planning and (iv) appraisal and feedback to help deliver goal directed approach.
	International Classification of Functioning, Disability and Health[6,22-24,138,149,153–157](11)	The International Classification of Functioning, Disability and Health describes functioning, disability and health using a framework of five components, representing an individual's body function and structure, activity, participation, environ- ment and personal factors.
	Canadian Occupational Performance Measure*[6,25–27,101,103–114,142,171,175,176] (21)	"The Canadian Occupational Performance Measure is an individualized measurement to detect change in client's satisfaction and performance in the three areas of self-care, productivity and leisure. Scoring scales ranges from 1, meaning "not able to do it" or "not satis ed at all", to 10, which is "able to do it extremely well" or "extremely satisfied".
	Goal Attainment Scaling*[28–57,113,114,118–143,150,172,181] (61)	Goal attainment scaling is a methodology that has been validated for measuring goal achievement individuals. Each goal is rated on a 5-point scale: +2 = much more than exptected; +1 = somewhat more than expected; 0 = Patient achieves the expected level; -1 = somewhat less than expected; -2 = much less than expected. Overall score is calculated by incorporating the goal outcome scores into a single aggregated t-score.
	Aid for Decision-making in Occupation Choice in English[58,100,101] (3)	The 'Aid for Decision-making in Occupation Choice', is an iPad application that uses text and images to assist patients to identify meaningful activities and social roles in their lives facilitating shared-decision making through the discussion between a therapist and a client.
	Patient-Specific Complaints instrument[59,60] (2)	The Patient Specific Complaints is a stepwise instrument for selecting and evaluating patient's main problems. In step one, the physiotherapist identifies which of the patient's daily activities are difficult to perform. Steps two and three involve prioritizing the activities and scoring them on a Numeric Rating Scale
	Patient Specific Goal setting method[61] (1)	The Patient Specific Goal setting method is a method to support physiotherapy goal setting. The method consists of six steps as follows. (1) Identifying problematic activ- ities that the patient encounters in daily life as a consequence of their health problem. (2) Prioritizing the most important activity he/she wants to work on in therapy. (3) Scoring the perceived ability to perform the selected activity on an 11-point scale. (4) Setting goals, i.e., translating the selected activities into treatment goals. (5) Planning treatment, i.e., making a shared decision about the treatment plan. (6) Evaluating the treatment goals.
	Talking Mats[62,157,175] (3)	Talking Mats is a strategy that allows persons of all ages who require intervention to express thoughts or emotions about specific topics through an easy-to-use visual framework. Graphic symbols are used as the means through which they are provided with a simple, yet powerful way of thinking about views in an effective, non-threatening way before expressing them.
	Collaborative Goal Technology[63] (1)	The Collaborative Goal Technology is an individualized goal striving intervention to enhance clinical practice and helping facilitate clinicians and people in recovery to collaboratively monitor goal progress at an individual and group level together. The overall objective of the Collaborative Goal Technology is to assist people with a psychiatric disability progress with their individual recovery processes.
	GOALS[64] (1)	GOALS consists of 24 items that evaluate life goals corresponding to six categories including intimacy (e.g. to have a close relationship), affiliation (e.g. to spend a lot of time with other people), altruism (e.g. to act unselfishly), power (e.g. to have a high social status), achievement (e.g. improve my skills continuously), and variation (e.g. to live an exciting life). The items pertaining to the different life goal categories were evaluated using 5-point rating scales to determine the importance (1 not important to 5 very important), attainability (1 very difficult to attain to 5 very easy to attain), and success in realizing a goal (1 not successful to 5 very successful).
	Improvement Scaling (Rehabilitation version)[65] (1)	Improvement Scaling (Rehabilitation Version) consists of 65 standardized goal scales representing the usual treatment goals pursued for rehabilitation patients. The scales are grouped into functional domains (eg, ambulation, balance, cognition, self- care, transfers, etc.). Each thermometer-like scale represents a continuous range of functioning, with descriptive phrases providing mean- ing to each region of the scale. Improvement Scaling appears to be a practical, reliable, valid, and clinically useful tech- nique for measuring individual patient change.
	Self-Assessment Goal Achievement[66,67,139] (3)	The Self-Assessment Goal Achievement questionnaire was developed to identify treatment goals and assess goal-achievement in patients with lower urinary tract symptoms. This information may promote patient-physician interaction and help patients establish realistic treatment goals, which may in turn improve treatment adherence and outcomes.
	The Goals for Occupational Therapy List[68] (1)	The Goals for Occupational Therapy List was designed for clients to self-identify goals for their outpatient rehabilitation. The Goals and Satisfaction Rating was designed to be paired with the Goals for Occupational Therapy List and measure the client's perception of goal achievement for the specific goals chosen on the Goals for Occupational Therapy List. Total scores ranging from 3 to 15 points were calculated for goal achievement by summing the scores of the three chosen goals.
	Patient Goal Priority Questionaire[69,70] (2)	Patient Goal Priority Questionaire has an open-ended format that initially asks patients to list 1 to 3 activities they are 1) unable or have difficulties performing due to pain, and 2) expect to improve as a result of PT. The patients estimate the relative importance of the activities by ranking them from 1 to 3, where 1 represents the most important activity to the individual. Three 11-point numerical rating scales follow each prioritized goal. For each goal, patients score current behavioral performance, satisfaction with current beha- vioral performance, and expectations of future behavioral performance (a-c). The newly developed measure Patient Goal Priority Questionaire with its patient-specific properties, has potential to serve: (a) as a clinical tool for identification of individuals' priorities of behavioral goals; (b) as a clinical tool for collaborative, formative eva- luation during the process of treatment, and (c) as a complementary self-report measure in research to assess clinically significant changes related to behavioral performance.
	Taxonomy for goals set in community-based acquired brain injury rehabilitation[97] (1)	The goal taxonomy has application and utility, at the individual client level and at the organizational level. It provides a basis for investigating goal setting and client concerns, and for enhancing the evaluation of community rehabilitation service delivery, particularly for people with acquired brain injury. It enables researchers and clinicians to develop a profile of issues of concern as identified by clients in a community rehabilitation setting.
	Goal setting tool to identify "Global meaning" of the client[98,99] (2)	The tool consists of the three steps: Exploring global meaning; Setting of the meaningful overlall rehabilitation goal; Setting and adjusting of specific rehabilitaiton goals. The tool defines the role of the client, rehabilitation physi- cian, chaplain and other members of the rehabilitation team in setting rehabilitation goals.
	Patient-Specific Functional Scale[9] (1)	Patient-Specific Functional Scale is a tool for problem identification evaluation. The response scale is 0 - 10.
	McMaster Toronto Arthritis Questionnaire[9] (1)	Aim of McMaster Toronto Arthritis Questionnaire is for identification of limitations owing to disease evaluation. This can be used for (1) Semistructured interview (2) Health assessment questionnaire among people with rheumatic and musculoskeletal diseases. Scoring domain includes activity participation. Response scale is yes-no visual analogue scale Likert scale.
	Problem Elicitation Technique[9] (1)	Aim of Problem Elicitation Technique is for identification of limitations owing to disease evaluation. This can be used for (1) Semistructured interview (2) Health assessment questionnaire among people with rheumatic and musculoskeletal diseases. Scoring domain includes self-care, mobility, role activities, social activities, emotion, communication, sleep and appearance. Response scale is Likert scale (1-10. Numerical Rating Scale, 0-10 Numerical Rating Scale,).

	Patient Goal Priority Questionnaire[9] (1)	Aim of Patient Goal Priority Questionnaire is to assist with problem identification, treatment goal selection, and evaluation in an interview setting for people with musculoskeletal pain using 0-10 or 0-4 numerical rating scale.
	Patient Goal Priority List[9] (1)	Aim of Patient Goal Priority List is to assist with problem identification, treatment goal selection and prioritization, and evaluation in an interview setting for people with musculoskeletal pain using 0-10 or 0-4 numerical rating scale.
	Patient-Specific Index[9] (1)	Patient-Specific Index is a questionnaire to assist with identification of the patients with total hip and knee arthroplasty' complaints and evaluation. This targets body function domain for scoring using Likert scale.
	Goal Facilitation Inventory[147] (1)	"This measure consists of 26 higher order life goals. Participants first evaluated the importance of each goal in their everyday lives on a 5-point Likert scale ranging from 'not at all important' (1) to 'very important' (5). Scores on these items were summed to obtain a goal importance score. On a scale ranging from 1 ('not at all hindered') to 5 ('completely hindered'), participants then reported the extent to which they were currently hindered in attaining each goal as a result of their amputation. Finally, a goal disturbance score was calculated by multiplying the goal impor- tance score for each item by its goal hindrance score and summing the resulting 26 product scores. This instrument demonstrates good internal consistency."
	Goal setting tip sheet[148] (1)	The goal setting tip sheet for the TASK III program was created from self-efficacy theory based on Bandura's and Lorig's work in self-management. The three main components of the skill-building goal setting tip sheet included: 1) Prioritizing acute and chronic health conditions, symptoms, and health habits; 2) Setting goals to improve health; and 3) Creating an action plan to meet health goals.
	Goal Pursuit Questionnaire[151],[152] (2)	"To assess a participant's habitual goal pursuit, a 24-item Goal Pursuit Questionnaire was developed. The items were drafted by the authors on the basis of the Mood-as-Input model. The items were construed such that they mea- sured the 2 Mood-as-Input model goals, hedonic and achievement goals, postulated to have opposing effects on behavior dependent on mood. Each item comprised a description of a daily situation in which hedonic goals conflict with achieve- ment goals. The Goal Pursuit Questionnaire included 24 vignettes belonging to 1 of 3 categories: a painful situation (8 items), an unpleasant nonpainful situation (8 items), and a pleasant nonpainful situation (8 items). The items on painful situations measured a person's preference for pain-avoidance goals relative to achievement goals. The items on nonpainful pleasant and unpleasant situations measured a per- son's preference for mood-management goals (ie, maintaining a positive mood) relative to achievement goals "
	Life Interests and Values Cards[158],[179] (2)	The Life Interests and Values Cards are comprised of 121 5 by 8 inch cards. All cards are black and white line-drawings, except the two "yes" and "no" cards used to support decision-making. Ninety-five cards picture a wide range of activities relevant to adults of all ages. Eleven cards depict feeling/emotions (e.g., happy, lonely) to be used with a visual analog so that the people with aphasia can indicate the degree to which they are experiencing those feelings and emotions. Seven cards show use of adaptations (e.g., adult tricycles) to indicate alternative ways of engaging in activities. Six composite picture cards indicate the categories for consideration by the people with aphasia.
	SMARTER[172] (1)	SMARTER stands for goals that are; S: Specific (well-defined and targets a specific problem to be addressed) M: Measurable (either quantitatively, as for technical goals, or qualitatively as for symptom-directed goals); Meaningful (achievement of goal should be beneficial to the patient or caregiver) A: Agreed upon (the patient or caregiver and clinician work toward a common end) R: Realistic (will the patient's potential for improvement and available resources support achievement of treatment goal?) T: Time-bound (achievement of goal should be within a reasonable amount of time) E: Evaluated (at predetermined points in time, goal achieve- ment and progress in doing so should be performed to deter- mine effectiveness of intervention) R: Revised (based on evaluation of goal achievement, new treatment goals may be identified, or prior ones revised)
	Needs Assessment Checklist[180] (1)	the Needs Assessment Checklist, which is a checklist of 216 behavioural indicators across nine rehabilitation domains: activities of daily living, skin management, bladder manage- ment, bowel management, mobility, wheelchair and equipment, community preparation, discharge coor- dination and psychological issues. The questionnaire uses a 4-point Likert scale ranging from 0 to 3 to assess the level of independence on each indica- tor with 0 representing 'completely dependent on staff', 1 representing 'mostly dependent on staff', representing 'moderately independent' and 3 repre- senting 'completely independent'. Item scores for each subscale are totalled and a 'percentage achieved score' is derived using a conversion table which reflects the patient's level of independence in each rehabilitation domain. Each Needs Assessment Checklist subscale total score ranges between 0 and 100% with higher Needs Assessment Checklist scores indicating greater level of independence. The questionnaire has strong internal con- sistency, item validity, test–retest reliability and concurrent validity.
	Client's Intervention Priorities[71] (1)	The Client's Intervention Priorities is a person-centered tool for defining rehabilitation priorities according to self-perceived functioning. The use of the Client's Intervention Priorities tool is encouraged to promote self-determination and optimal involvement of individuals with acquired brain injury in interdisciplinary neurorehabilitation. With the Client's Intervention Priorities tool, individuals are asked to judge their degree of functioning in 41 everyday/life situations corresponding to six categories representing daily activities (21 items: nutrition, fitness, personal care, communication, housing, mobility) and six categories representing social roles (20 items: responsibilities, interpersonal relationships, community life, education, employment, recreation).
	Framework for describing goals and content of exercise interventions[72] (1)	a framework to describe goals and content of exercise interventions in physical therapy. Step 1. Select the most common intervention goals, with a maximum of five goals on the level of body functions and a maximum of five goals on the level of activities and participation. Describe the goals in The International Classification of Functioning, Disability and Health terminology (see list of goals below). Step 2. Give a detailed description of the intervention using the list of intervention details as a guide (see list of intervention details below). Step 3. Number the exercises within the intervention and link them to the goals they are related to. Step 4. Summarize common information concerning exercises under 'General information'. Step 5. Insert a timeline when applicable: if different phases in the intervention period can be distinguished, intervention goals that are applicable for a particular phase should be described per phase.
	The Goal setting and Action Planning[73],[74],[116],[117],[149] (5)	The Goal setting and Action Planning framework guides patient centred goal setting practice in community stroke rehabilitation settings. Evidence and theory based, the Goal setting and Action Planning framework informs a collaborative approach between stroke survivors and staff to the setting and pursuit of rehabilitation goals. It has four key stages: (i) goal negotiation and setting, (ii) action planning and coping planning, (iii) action and (iv) appraisal, feedback and decision making. Stroke survivors personal goals, plans and appraisals are recorded in the stroke survivor held the Goal setting and Action Planning record.
	Person Environment Profile[75] (1)	The Person Environment Profile is a new instrument to assist with understanding the perceived role of personal and environmental factors in the attainment of rehabilitation goals. The Person Environment Profile helps identify the client's perspective of both environmental and personal factors, which help or hinder the attainment of a particular goal. The Person Environment Profile explores 10 elements: five personal and five environmental. The five personal elements are personality, health and fitness, problem-solving ability, motivation, and attitude. While the five environmental elements in the Person Environment Profile are derived from the environment chapters of the International Classification of Functioning, Disability and Health terminology, they do not encompass the full taxonomy of the International Classification of Functioning, Disability and Health terminology.
	The Self-Management Goal Cycle framework[76] (1)	The Self-Management Goal Cycle framework illustrates a model of care for patients with diabetes. In the framework, collaborative goal setting is a valuable tool for improving self-management skills among patients with diabetes. By implementing goal setting techniques, members of the patient care team are better equipped to help patients manage their chronic conditions by making them valued partners of the health care team.
**n=15)**	Goal Management training*[77–86],[144–146] (13)	Each of the five Goal Management training stages corresponds to an important aspect of goal-directed behavior. In Stage 1, orienting, participants are trained to assess the current state of affairs and direct awareness towards relevant goals. Goals are selected in Stage 2, and these are partitioned into sub- goals in Stage 3. Stage 4 concerns encoding and retention of goals and subgoals. In Stage 5, the outcome of action is compared with the goal state (monitoring). In the event of a mismatch, the entire process is repeated.

Tools for goal setting and intervention delivery (	the FOURK model[87] (1)	The FOURC model can help the collaborating client–clinician team to identify meaningful goals by the end of the first session, effective intervention planning is always distributed over many sessions. The planning begins at the time of the initial evaluation and continues throughout the rehabilitation program, melding with intervention strategies, further assessments, observations, and conversations to become an integral component of a comprehensive aphasia rehabilitation program. The four steps are (1) choose a communication goal, (2) create client solutions, (3) collaborate on a plan, and (4) complete and continue. The purpose of this clinical focus article is to present a step-by-step model for forming a collaborative partnership with clients to develop an intervention plan that follows the client's lead, addresses communicative participation, and integrates multiple treatment strategies.
	Dietary behaviour change tool*[96] (1)	The structure of the online diet goal-set- ting tool was informed by online computer-based diabetes self-man- agement goal-setting tools16 and the 4 major steps of dietary behavior change: 1. Dietary Assessment; 2. Goal setting; 3. Goal Action Plan; 4. Goal Self-Monitoring
	Cognitive Orientation to daily Occupational Performance[102,103] (2)	Cognitive Orientation to daily Occupational Performance, the top down task-oriented, individualized approach originally developed for children with developmental coordination disorders in 2001. During a Cognitive Orientation to daily Occupational Performance intervention, therapist guides the client in learning of this self-instruction strategym which enables him/her to identify which part of the performance is wrong, and to invent and execute plans to correct his/her task performanec by using the goal-plan-do-check strategy.
	Identity Oriented Goal Training[114,144,178] (3)	In this approach, the intervention is more 'top down', primarily aiming to enable the client to identify a meaning- ful, higher representation of what is important to them as a basis for driving their goals. This is supposed to enhance motivation, mood and goal performance. Identity Oriented Goal Training is the use of metaphor and identity mapping in order to facilitate such engagement. In this approach, an identity map as a tool for inter- vention delivery and scripted a six-step process for clinicians to follow throughout the course of the intervention period to aid the iden- tity mapping process . Step 1: Talk with the client about things they might like doing or being involved in and identify someone who they admire; step 2: talk with client about the facts of that individual; step 3: talk with client about the appearance of that individual; step 4; talk with client about what that individual achieves and might aim for - the goals of that individual; step 5: talk with client about how it would feel to achieve things that they admire - how would it feel to achieve those goals; step 6: talk with client about steps they might take to move towards those goals - strategies and actions
	Functional Goal-setting and Self-management tool[115] (1)	The Functional Goal-setting and Self-management tool includes information about osteoarthritis pathophysiology and management, discussion of pain and impaired function, and activity and goal setting sections to help establish and track functional goals.
	Bern Invenstory of Treatment Goals[143] (1)	Bern Inventory of Treatment Goals, a previously published goal taxonomy developed for psychotherapy goals.
	Occupational Goal Intervention*[159–161] (3)	The Occupational Goal Intervention program focuses on strategy learning using activities and everyday tasks. The steps of the program followed Goal Management Training but with a focus at the beginning on the individual choice of meaningful activities and at the end on debriefing of the activity performance. The treatment process emphasized the use of functional activities in three main domains: (1) food preparation; (2) money management; and (3) reading, writing, and using computers for in-formation seeking. However, the functional domain was adapted to each client's choices and needs when the client preferred to work on other activity domains. The as- sumption is that the learned thinking process is trans ferred to any other occupational performance domains
	The client-centred goal setting practice framework[17,176] (2)	This theoretical framework is to explain how clinicians support clients with TBI to actively engage in goal setting in routine practice by utilizing three phases: (1) a needs identification phase (2) a goal operationalisation phase; (3) an intervention phase. "According to the framework, client-centred goals are developed during three phases: a 'needs identification phase', a 'goal operationalisation phase' and an 'inter- vention phase'. The three phases are represented by five broad processes. The initial needs identification phase incorporates the synchronous processes of 'establishing trust' and 'identifying the person's needs'. Next, the goal operationalisation phase includes the 'goal map- ping' process or when rehabilitation needs are unable to be identified, clients are engaged in the 'allowing time' process. Lastly the intervention phase encompasses the process of 'active engagement'. In addition to the processes which occur during each phase, each process is represented by strategies. Strate- gies describe the numerous techniques used by practi- tioners to implement the five broad processes of the framework."
	The Goal Sharing for Partners Strategy[177] (1)	The Goal Sharing for Partners Strategy is a tool for audiologists to use with patients with hearing loss and their communication partners. This strategy provides a framework that is designed to assist both the patients with hearing loss and their communication partners to: (1) acknow- ledge the hearing loss; (2) acknowledge the hearing loss-related activity limitations and participation restrictions that they each face as a result of the hearing loss; (3) acknowledge that they are partners in communication; and (4) develop an understanding of their shared responsibility in dealing with the hearing loss. The Goal Sharing for Partners Strategy is a shared goal-setting strategy that enables patients with hearing loss and their communication partners to develop realistic communication goals and to consider the steps necessary to achieve these goals.
ols for measuring the quality of goal setting (n=9)	the Client-Centeredness of Goal Setting Scale[88,176] (2)	Client-Centeredness of Goal Setting Scale as a tool to evaluate and promote client-centered goal planning in brain injury rehabilitation. The Client-Centeredness of Goal Setting Scale was developed to promote and enhance client-centered goal planning through greater understanding of the client's perspective on planning processes and the resultant goals. The Client-Centeredness of Goal Setting Scale scale is intended to be administered as soon as possible after goal planning is complete and rehabilitation goals are documented.
	Goal-Setting Evaluation Tool for Diabetes[89] (1)	The Goal-Setting Evaluation Tool for Diabetes, a criteria-based, observer rating scale that measures the quality of patients' diabetes goals and action plans. The Goal-Setting Evaluation Tool for Diabetes can reliably and validly rate the quality of goals and action plans. It holds promise as a measure of intervention fidelity for clinical interventions that promote diabetes self-management behaviors to improve clinical outcomes. The Goal-Setting Evaluation Tool for Diabetes is to assess quality of goals and action plans for people with diabetes.
	Goal Engagement Scale[90] (1)	the Goal Engagement Scale examines patients', families' and health professionals' perceptions of patient engagement in goal setting. Each participant was asked to complete the scale using the following instructions: i) circle the appropriate descriptor to rate the patient's level of engagement in goal setting; ii) classify who the patient perceived to be mainly involved in setting goals. The Level of Engagement in Goal Setting Scale ranged from a rating of 0 to 5: Unable; Minimal engagement (1); Moderate engagement (2); Good engagement but requires active support (3); Very good engagement requiring low-level support (4) Excellent engagement (5). Classifications of who was mainly involved in goal setting included: Goals set mainly by patient; Goals set mainly by family; Goals set mainly by health care team; Goals set mainly by family and health care team together; Goals set mainly by patient and health care team together; Goals set jointly by patient, family and health care team.
	The Goal Instrument for Quality[91] (1)	The Goal Instrument for Quality was developed based on these goal-setting principles and provides a numerical score for goal quality. Goal Instrument for Quality Items assess following items: 1.Vision 2.Collaboration 3.Behaviorally defined goals 4.Goal importance 5.Confidence/self efficacy 6.Time frame for goals 7.Level of goals attainment 8.cton plans for goals 9.Identifying and problem solving barriers 10.Social support 11.Monitoring of goals. Ten of the 11 items are rated on a three-point scale (2 = completed, 1 = partially completed, 0 = no attempt) and one item (#2) is rated on 2-point scale (2 = complete, 0 = no attempt). A detailed description for each possible score and item is included to guide raters. An overall goal quality score can be obtained (a sum of all of the items scores) as well as individual item scores.
	The Seeking of Noetic Goals test[92,173] (2)	The Seeking Of Noetic Goals test, an attitude scale designed to measure the strength of the motivation to find meaning and purpose in life, to complement the purpose in life test. The Seeking Of Noetic Goals consists of 20 statements, each rated on a seven-point Likert-type scale ranging from1 (never) to 7 (constantly).
	The Purpose in Life[92,173] (2)	The Purpose In Life test is an attitude scale, which indicates the degree to which meaning and purpose in life have been attained by the respondents. It consists of 20 statements, each rated on a seven-point verbal scale with two anchoring phrases. Sample items read: "In life I have: no goals or aims; clear goals and aims"; "In achieving life goals I have: made no progress whatsoever; progressed to complete fulfillment." The total score, obtained as a sum across all items, ranges from 20 to 140, with higher scores indicating a clearer meaning and purpose in life.

Too	Goal Adjustment Scale[147] (1)	The Goal Adjustment Scale was completed at both time points. This scale consists of ten items assessing how respondents typically react if they have to stop pursuing an important goal in their life. Four items assess the tendency to disengage from unattainable goals; six items measure the capacity to re-engage in new goals. Items are rated on Likert scales ranging from 1 (‘strongly disagree’) to 5 (‘strongly agree’). This measure has acceptable reliability and validity. In the present study, Cronbach’s alpha values of 0.70 and 0.91 were observed for the disengagement and re-engagement subscales, respectively.
	Alberta Shared Decision-making Measurement Instrument[162] (1)	This tool is to gather information about shared decision making between the client and the client's health care provider. (7 likert scale for six questions)
	Patient-focused goal planning questionnaire[180](1)	This 36-item survey contained both quantitative and qualitative components and was developed by the spinal injury unit multidisciplinary team to measure the patient satisfaction, perceptions and experiences with the goal planning process. The quantitative responses obtained were rated on a 6-point Likert scale ranging from 1 to 6 with 1 representing ‘strongly agree’ and 6 representing ‘strongly disagree’. Additional space was provided within this questionnaire for qualitative comments and recommendations. This question- naire is available from the authors upon request.
